# A commentary on: ‘Preoperative carbohydrate loading with individualized supplemental insulin in diabetic patients undergoing gastrointestinal surgery: a randomized trial’

**DOI:** 10.1097/JS9.0000000000000407

**Published:** 2023-06-05

**Authors:** Deqing Chen, Yingxiao Zhang, Huanhuan Huang, Zijin Qiu

**Affiliations:** a Department of Endocrinology, The People’s Hospital of Rongchang District Chongqing, China; b Department of Geriatrics; c Department of Nursing, The First Affiliated Hospital of Chongqing Medical University; d Clinical Cosmetology Teaching and Research Office, Chongqing Medical and Pharmaceutical College, Chongqing, People’s Republic of China

*Dear Editor*,

Preoperative carbohydrate loading is a component of Enhanced Recovery After Surgery (ERAS) protocols and has been widely investigated among patients undergoing orthopedic procedures^1^. Administration of carbohydrate nutrition 2 and 12 h before surgery has been shown to decrease insulin resistance in the perioperative period without posing an increased risk for aspiration or other postoperative complications. Previous studies highlighted that preoperative carbohydrate loading could improve patient comfort after elective abdominal surgery by decreasing thirst, hunger, anxiety, malaise, and overall length of stay. Despite the known benefits of preoperative carbohydrate loading, there is limited research on diabetic patients undergoing gastrointestinal surgery. Li *et al*.^2^ conducted a randomized controlled trial to investigate the effects of preoperative carbohydrate loading using an individualized supplemental insulin regimen on diabetic patients undergoing gastrointestinal surgery. They concluded that preoperative carbohydrate loading with individualized supplemental insulin did not promote gastrointestinal recovery but improved per-operative well-being in diabetic patients undergoing gastrointestinal surgery.

Patients with diabetes mellitus are at a higher risk of developing postoperative impaired glycemic control and they run a greater risk of complications after surgery. Acute elevations in blood glucose concentrations are known to delay gastric emptying in both healthy subjects and patients with diabetes^3^. However, despite the association between glucose levels and gastric emptying rates, blood glucose levels per se have not been shown to be a reliable predictor of gastric emptying. Moreover, there is no medical evidence to show any association between peak glucose concentrations and gastric emptying rates. To implement proper guidelines, including the use of preoperative carbohydrate loading with individualized supplemental insulin as a routine for diabetic patients, it would be good if patients with reduced gastric emptying rates could be preoperatively identified. However, the correlation between gastric emptying rates and autonomic neuropathy or upper gastrointestinal symptoms is weak, and physical examination signs and laboratory tests are of little value.

In the ‘Results’ section, the authors reported that three patients in the carbohydrate group had protocol deviation as they only took the carbohydrate drink the night before surgery but not in the morning of surgery due to patients’ concerns about a full stomach. However, in their Table 3 (outcomes for all patients), the authors included these patients to compare the two groups. As these three patients are equivalent to patients undergoing routine preoperative fasting, if no carbohydrate drink was used on the day of surgery, they should have been excluded from the carbohydrate group.

We respectfully appreciate that Li *et al.* provided us with an important study focusing on the effects of carbohydrate loading with individualized supplemental insulin in diabetic patients undergoing gastrointestinal surgery. This study can provide a guide to clinicians for decision-making. However, more studies with large sample size and good scientific design should be carried out to clarify this issue.

## Ethical approval

Ethical approval is not required as the type of the manuscript is a letter.

## Sources of funding

Chongqing Science and Health Joint Medical Research Project (2022MSXM181).

## Author contribution

D.C.: drafted the manuscript; Y.Z.: revised the manuscript; H.H.: data analysis; Z.Q.: project support.

## Conflicts of interest disclosure

There are no conflicts of interest.

## Research registration unique identifying number (UIN)

None.

## Guarantor

Zijin Qiu is the guarantor.
